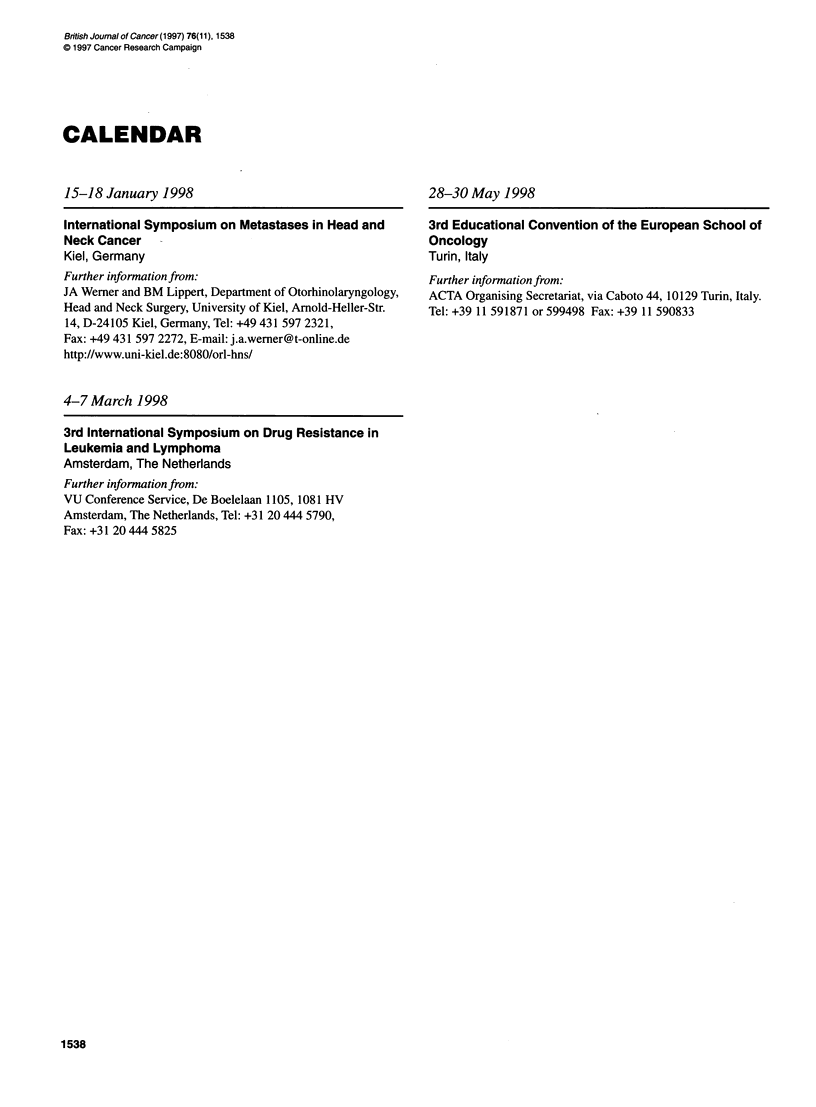# Calendar

**Published:** 1997

**Authors:** 


*British Journal of Cancer* (1997) **76**(11), 1538
© 1997 Cancer Research Campaign

### CALENDAR

*15–18 January 1998*

**International Symposium on Metastases in Head and Neck Cancer**
Kiel, Germany

*Further information from:*
JA Werner and BM Lippert, Department of Otorhinolaryngology, Head and Neck Surgery, University of Kiel, Arnold-Heller-Str. 14, D-24105 Kiel, Germany, Tel: +49 431 597 2321, Fax: +49 431 597 2272, E-mail: j.a.werner@t-online.de http://www.uni-kiel.de:8080/orl-hns/

*4–7 March 1998*

**3rd International Symposium on Drug Resistance in Leukemia and Lymphoma**
Amsterdam, The Netherlands

*Further information from:*
VU Conference Service, De Boelelaan 1105, 1081 HV Amsterdam, The Netherlands, Tel: +31 20 444 5790, Fax: +31 20 444 5825

*28–30 May 1998*

**3rd Educational Convention of the European School of Oncology**
Turin, Italy

*Further information from:*
ACTA Organising Secretariat, via Caboto 44, 10129 Turin, Italy. Tel: +39 11 591871 or 599498 Fax: +39 11 590833